# The role of mitochondrial-associated endoplasmic reticulum membranes (MAMs) in diabetic microvascular complications: a review

**DOI:** 10.1038/s41419-025-08236-1

**Published:** 2025-12-09

**Authors:** Yudian Wang, Yufei Zhang, Qi Jin, Hailing Zhao, Ping Li

**Affiliations:** 1 College of Traditional Chinese Medicine, Hubei University of Chinese Medicine, Hubei Wuhan, China; 2 Guang’anmen Hospital, China Academy of Chinese Medical Sciences, Beijing, China; 3 Institute of Clinical Medical Sciences, China-Japan Friendship Hospital, Beijing, China

**Keywords:** Molecular biology, Diseases

## Abstract

Diabetic vascular complications include macrovascular lesions and microvascular lesions. Diabetic microvascular complications (DMC) are mainly manifested by microvascular endothelial dysfunction, basement membrane thickening, capillary permeability changes and microthrombosis, which may contribute to the occurrence of kidney, cardiac, ocular and peripheral system damage in diabetic individuals. Thus, it is urgent to develop new prevention and treatment strategies. The mitochondria-associated endoplasmic reticulum membranes (MAMs), as a highly heterogeneous membrane contact site, play a key role in maintaining biological communication between the endoplasmic reticulum (ER) and mitochondria. Studies have shown that MAMs are involved in the pathogenesis of DMC by regulating Ca^2+^ homeostasis, lipid synthesis and transport, mitochondrial dynamics, ER stress, glucose homeostasis, autophagy, apoptosis, and inflammation. This review comprehensively summarizes the complex structure and key biological functions of MAMs that drive the physiological transmission of substances and signals between organelles. Furthermore, we focused on exploring the regulatory mechanism of MAMs on different diabetic microangiopathies, including diabetic kidney disease (DKD), diabetic cardiomyopathy (DCM), and diabetic retinopathy (DR). In conclusion, targeting MAMs is a promising but challenging therapeutic strategy.

## Facts and questions

- The mitochondria-associated endoplasmic reticulum membranes (MAMs), as a highly heterogeneous membrane contact site, play a key role in maintaining biological communication between the endoplasmic reticulum (ER) and mitochondria.
- MAMs are involved in a variety of physiological and pathological processes, including regulating calcium transport, lipid metabolism, mitochondrial function, ER stress, cell autophagy, and oxidative stress.
- MAMs dysfunction is a risk factor for the progression of several diseases, such as diabetic kidney disease (DKD), diabetic cardiomyopathy (DCM), and diabetic retinopathy (DR).
- Targeted MAMs for the treatment of diabetic microvascular complications (DMC) is a promising but challenging strategy.

## Introduction

Diabetes mellitus (DM) is a kind of metabolic disorder typified by hyperglycemia [1]. Long-term metabolic abnormalities in DM lead to chronic vascular complications, including macrovascular and microvascular lesions, which are the significant contributor causing disability and death in DM patients [2]. Diabetic microvascular complications (DMC) are the most common early complications of DM. An evaluation study in China showed that the prevalence of DMC was about 20% in DM patients with a disease duration of less than 1 year, and up to 50% in those with a disease duration of more than 10 years [3]. DMC is typically characterized by micro venous, capillary, and micro arterial lesions, resulting in pathological changes in various tissues and organs, such as kidney, retina, myocardium, and nerve tissues, which are respectively manifested as diabetic kidney disease (DKD), diabetic retinopathy (DR), diabetic cardiomyopathy (DCM), and diabetic peripheral neuropathy [4]. DMC is the key factor contributing to the decline in the quality of life and the shortening of life expectancy among DM patients, imposing a heavy burden on patients’ families and society [5]. As the global incidence of DMC increasing year by year, it is particularly urgent to explore the molecular mechanism of its occurrence and development [6]. At present, researchers are dedicated to exploring new targets for the treatment of DMC, aiming to pioneer effective intervention strategies.

Over the past few decades, the influence of organelles in human diseases has received extensive attention. Maintaining organelles in a state of homeostasis is imperative to the regular function of live cells. With the in-depth exploration of organelles, it is found that organelles do not exist and operate in isolation, and the interaction between organelles can foster signal transduction and material exchange, which may generate antagonistic or synergistic effects [7]. It has been shown that mitochondria can be coupled to other organelles like the endoplasmic reticulum (ER), Golgi apparatus, lysosomes, and the nucleus [8]. The mitochondrial outer membrane-ER junction is the earliest recognized and most detailed physical interaction between organelles, and the contact areas have been identified as mitochondrial-associated ER membranes (MAMs) [9]. MAMs are dynamic structure that can rapidly respond to functional changes within cells and serves as a crucial hub for intercellular communication [10]. Recent studies have indicated that MAMs are involved in a variety of physiological and pathological processes, including regulating calcium transport, lipid metabolism, mitochondrial function, ER stress, glucose homeostasis, autophagy, apoptosis, and inflammation [11, 12]. Researchers emphasize that MAMs dysfunction is a risk factor for the progression of several diseases, such as DM, DKD, DR, diabetic encephalopathy, cancer, cardiovascular diseases, and neurodegenerative diseases [13–17]. Recently, the mechanism of MAMs in diabetic microangiopathy is continuously deepening. Therefore, this review comprehensively introduces the molecular characteristics and key physiological functions of MAMs, focuses on discussing its role in three types of diabetic microangiopathy: DKD, DCM, and DR, and finally explores the possibility of MAMs as a potential target for the treatment of DKD, DCM, and DR.

## The morphologic structure and molecular composition of mams

In 1965, the specialized region between the mitochondria and ER was initially discovered by William Bernhard applying an electron microscope [18]. By the 1990s, Vance isolated this unique structure from rat livers using subcellular grading technology, thereby proposing the term “MAMs” and simultaneously discovering the transfer of lipids in the site of MAMs [19]. The existence of MAMs was subsequently verified with advanced imaging techniques, for example, fluorescent proteins, real-time imaging, and high-resolution imaging techniques [20, 21]. MAMs is dynamic rather than fixed. MAMs can be categorized morphologically into three types considering the size of the contact area [22]: (I) The first type is the common small-area contact type, where the ER tubules typically extend toward the mitochondria, and the contact surface roughly covers 10 percent of the mitochondrial surface; (II) The second type is covering approximately half of the mitochondrial surface; (III) The third type is that the ER tubules enclose the whole mitochondria, forming a complete surrounding structure. As observed by electron tomography, the spatial gap of MAMs stayed within the range of 10 and 30 nanometers, which is the optimal condition for maintaining efficient protein interaction [23]. Notably, the distance between the mitochondria and ER might vary somewhat based on the cell state to adapt to different microenvironments. For example, the MAMs distance in the 10 to 25 nm range contribute to Ca^2+^ exchange [24]; The ER is tightly merged with mitochondria to form a contact area smaller than 10 nm, which is appropriate for lipid transfer [25]; If the ER tubules envelop mitochondria at a length of roughly 30 nm, the process of mitochondrial fission is facilitated [26]. A recent study has shown that knocking down Zdhhc9 and silencing protein kinase G1 (PKG1) can alter the activity of MAMs resident proteins and the physical distance of MAMs in osteoblasts [27]. Consequently, the ER-mitochondria distances that are outside of the standard physiological range can cause dysfunction in MAMs.

**Fig. 1:**
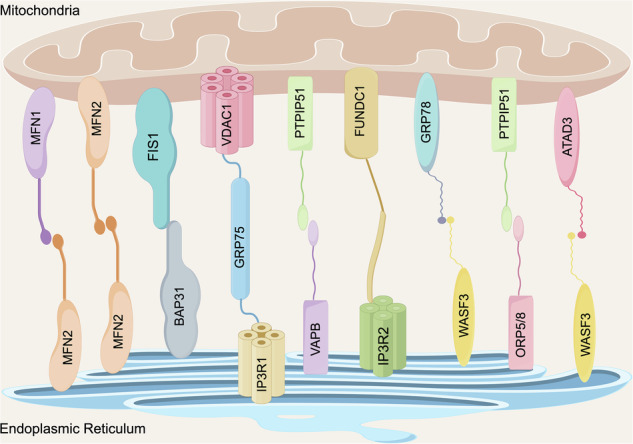
The morphologic structure and molecular composition of MAMs. The specialized region between the ER and mitochondria is called MAMs, and this structure is connected by some protein complexes. MFN1/2: mitofusin 1/2; BAP31: B-cell receptor-associated protein 31; FIS1: fission 1; IP3R1: inositol 1,4, 5-triphosphate receptor 1; GRP75: glucose-regulating protein 75; VDAC1: volt-dependent anion-selective channel 1;VAPB: Vesicle-associated membrane protein-associated protein B; PTPIP51: protein tyrosine phosphatase interacting protein 51; IP3R2: inositol 1,4, 5-triphosphate receptor 2; FUNDC1: FUN14 domain-containing protein 1; WASF3: Wiskott-Aldrich syndrome protein family member 3; GRP78: glucose-regulating protein 78; ORP5/8: oxysterol-binding protein-related protein 5/8; ATAD3: ATPase family AAA Domain-containing protein 3.

By employing proteolytic hydrolysis to isolate MAMs, Csordas et al. originally revealed that the core component between the ER and the outer mitochondrial membrane (OMM) is anchoring protein complex [23]. Thus far, over 1000 proteins linked to the structure and function of MAMs have been identified in tissues such as the liver, testicles and brain of mice applying proteomics technology [28–30]. These proteins are mainly classified into the following three categories: (1) the proteins are specifically present only in MAMs; (2) the proteins enrich concurrently in MAMs and other organelles; (3) It temporarily exists in MAMs under specific circumstances [31]. It is worth noting that the protein composition of MAMs will alter in accordance with the status of proliferation and apoptosis in cells. A comprehensive proteomic analysis on MAMs in the brains of diabetic and non-diabetic mice showed that 144 proteins underwent significant changes during exposure to high glucose [32]. As crucial linkages between the ER and mitochondria, MAMs related proteins flexibly carry out functional regulation. The key anchoring protein complexes of MAMs in mammalian cells include: (a) The IP3R1-GRP75-VDAC1 complex is made up of inositol 1,4, 5-triphosphate receptor 1 (IP3R1) on the ER membrane and volt-dependent anion-selective channel 1 (VDAC1) on OMM. VDAC1 and IP3R1 get linked via the molecular chaperone glucose-regulating protein 75 (GRP75) in order to stably sustain Ca^2+^ transport [33]. (b) The mitochondrial fission 1 and the B-cell receptor-associated protein 31 existing on the ER combine to form the BAP31-FIS1 complex, which plays a role in apoptosis signaling and mitochondrial dynamic equilibrium [34]. (c) Mitochondria-shaping mitofusin 2 (MFN2) participates in mitochondrial Ca^2+^ absorption and autophagosome formation by establishing heterotypic and homotypic complexes with mitofusin 1 (MFN1) or MFN2 [35, 36]. (d) One unique bridge between OMM and the ER is formed by FUN14 domain-containing protein 1 (FUNDC1) and IP3R2, which regulates the movement of Ca^2+^ to mitochondria and sustains mitochondrial dynamics [37]. (e) Vesicle-associated membrane protein-associated protein B (VAPB) enriched in the ER and mitochondrial-resident protein tyrosine phosphatase interacting protein 51 (PTPIP51) interact to constitute the VAPB-PTPIP51 complex to assist with the transport and balance of Ca^2+^ [38]; Furthermore, PTPIP51 can also attach to oxysterol-binding protein-related protein 5/8 (ORP5/8) to encourage the transport of phosphatidylserine [39]. In addition to the above, there are other protein complexes in MAMs involved in biological processes, as shown in Fig. 1. Nevertheless, the proteins identified so far may only represent a small fraction of the entire MAMs proteome, and further comprehensive analysis of proteins between the ER and mitochondria is required.

## Key biological functions of MAMs

### Ca^2+^ homeostasis

**Fig. 2:**
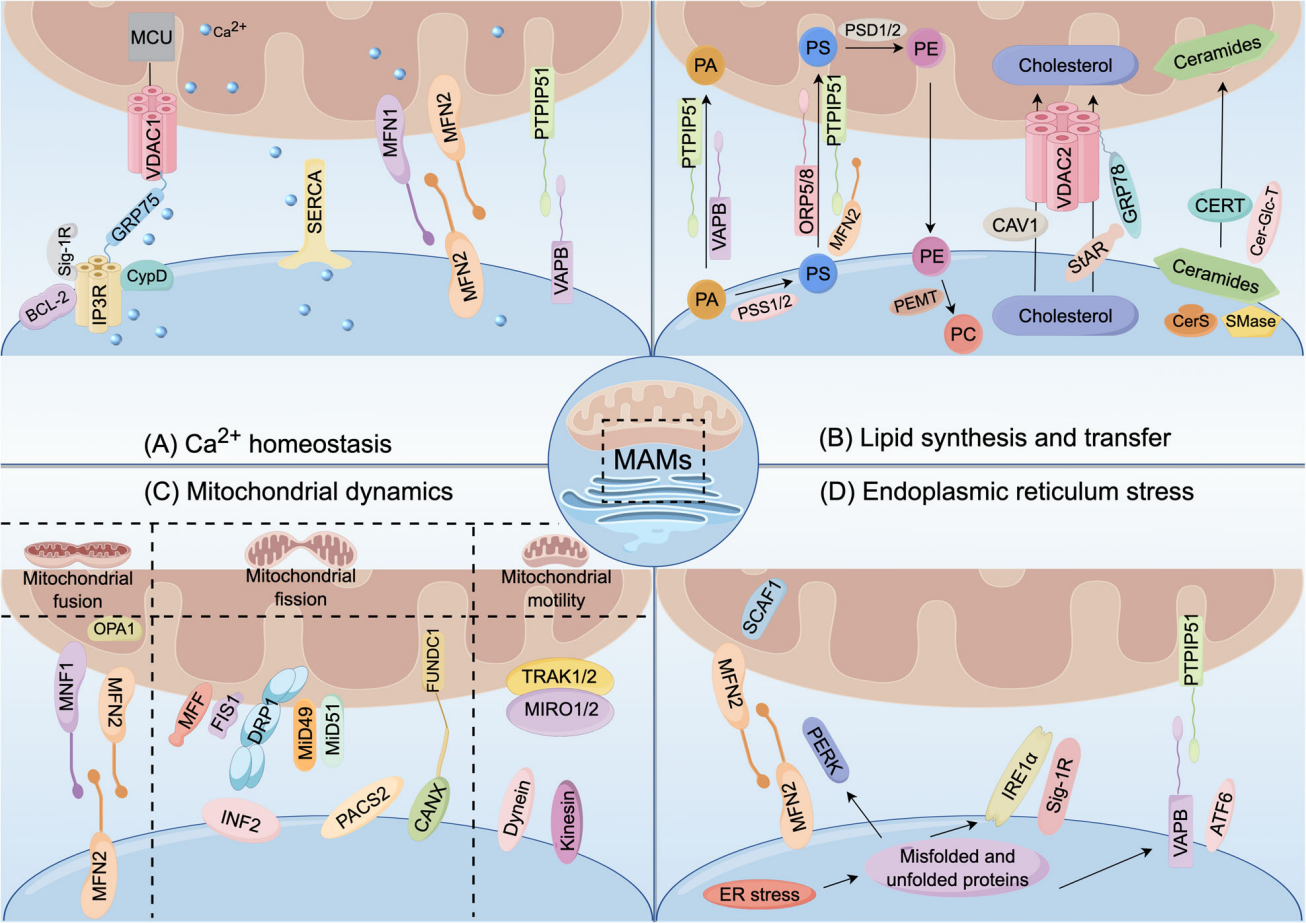
The biological functions of MAMs. This figure illustrates the biological functions of MAMs, including (**A**) Ca^2+^ homeostasis; **B** lipid synthesis and transfer; **C** mitochondrial dynamics; **D** ER stress.

Ca^2+^ act as a second messenger regulating various cellular processes, and its flux imbalance has been tied to multiple human diseases [40, 41]. The homeostasis of Ca^2+^ is primarily manifested as the exchange and balance of Ca^2+^ between the mitochondria and ER, which is a crucial signal for maintaining the normal function of cells [42]. MAMs be used as the hubs for Ca^2+^ signal transduction. Both the mitochondria and the ER are key organelles for storing Ca^2+^, and the Ca^2+^ transfer from the ER to the mitochondria is dependent on some contact sites mediated by MAMs, as depicted in Fig. 2A [43]. For cell survival and the generation of bioenergy, the Ca^2+^ oscillation of mitochondria is indispensable. Under physiological conditions, mitochondrial Ca^2+^ regulates vital enzymes in the tricarboxylic acid (TCA) cycle, including isocitrate dehydrogenase, pyruvate dehydrogenase, and α -ketoglutarate dehydrogenase, to generate ATP [44]. Yet, overload of Ca^2+^ in the mitochondria can activate the opening of mitochondrial permeability transition pores (MPTP), triggering the apoptotic procedure [45]. It has been found that mitochondrial Ca^2+^ transport and MAMs parameters, like coupling number, coupling expansion, and MAMs length, are inversely associated [23]. An increase in Ca^2+^ transport efficiency with shortening the distance between MAMs is likely to result in overload of mitochondrial Ca^2+^. The effectiveness of Ca^2+^ transport declines with increasing the distance. Interestingly, Csordas et al. found that when the gap between mitochondria and ER is shorter than 7 nm, due to the inadequate space of IP3R, the transport efficiency of Ca^2+^ exhibits a significant downward trend, indicating that the appropriate MAMs distance is fundamental for ensuring Ca^2+^ transport [46].

After the cell is stimulated, the ER, the largest intracellular calcium storage pool, releases Ca^2+^ into the cytoplasm to deliver calcium signals [47]. Roughly, after being released from the ER, Ca^2+^ passes through two layers of mitochondrial membranes, namely the OMM and the inner mitochondrial membrane (IMM), and is subsequently pumped back into the ER [48]. In this process, the effective transport of Ca^2+^ is mediated by multiple resident protein complexes. IP3R1, the primary channel protein for Ca^2+^ release in the ER, is in charge of setting up a high Ca^2+^ zone around the ER [49]. IP3R is classified into three subtypes, and the majority of cells carry more than one subtype [50]. IP3R1 and IP3R2 are more typical in hepatocytes, whereas IP3R3 is more predominant in cholangiocytes [22, 51]. By ligating the molecular chaperone GRP75, IP3R collaborates with the high-conductivity protein VDAC1 located in the OMM and facilitates the uptake of Ca^2+^ by the OMM [52]. However, since the IMM is impermeable to ions, the inflow of Ca^2+^ into the mitochondrial matrix through the IMM is accomplished by an electrical gradient via mitochondrial calcium uniporter (MCU) [53]. MCU is a complex composed of MCU1, MCU2, MICU1, MICU2, EMER, and so on [54]. MICU1/2 is a Ca^2+^ uptake regulating protein that serves as an instrument for sensing Ca^2+^ concentration between membranes. MICU1/2 is suppressed at low cytoplasmic Ca^2+^ level. However, MICU1/2 acts as a gating tool to avoid mitochondrial Ca^2+^ overload when the Ca^2+^ concentration reaches the threshold [55, 56]. The above indicates that IP3R-GRP75-VDAC1 is the Ca^2+^ transfer channel through MAMs. When GRP75 is knocked down, the physical link between VDAC1 and IP3R becomes unstable, affecting the transfer efficiency of Ca^2+^ [33]. Other molecular chaperones found in MAMs, for example, cyclosporine D (CypD), is a Ca^2+^ sensitive mitochondrial folding enzyme that can regulate the spatial structure of MAMs and interact with the IP3R-GRP75-VDAC1 protein complex at the MAMs region to transfer Ca^2+^ from the ER to mitochondria [57]. Additionally, the Ca^2+^ ion flow is managed by the ER-resident protein SERCA, which actively pumps Ca^2+^ from the cytoplasm into the ER, thereby forming a Ca^2+^ gradient in the cytoplasm-ER to sustain appropriate Ca^2+^ concentrations in the ER and considerably contributing to mediating Ca^2+^-induced apoptosis [52]. Typically, the SERCA is encoded by three genes, namely SERCA1, SERCA2 and SERCA3. The variant of SERCA1(S1T) is a protein truncated from the C-terminal, whose function is to prevent the pumping of Ca^2+^, resulting in the leakage of Ca^2+^ in the ER. Under pathological conditions, S1T is triggered to enhance Ca^2+^ transport from the ER to mitochondria and is involved in the process of Ca^2+^-dependent mitochondrial apoptosis [58]. Researchers have discovered that MFN2 in the ER comes into contact with MFN2/ MFN1 in OMM, forming bridges across organelles and influencing Ca^2+^ flow. But there is still controversy over precise function of MFN2, as different research teams have produced inconsistent results about its role in calcium transport [59]. Besides, recent studies have also reported a large number of related molecules residing in the MAMs region that are involved in regulating calcium transport, such as VAPB-PTPIP51, B-cell lymphoma 2 (BCL-2), and sigma-1 receptor (Sig-1R) [60–62].

### Lipid synthesis and transfer

Lipids are active molecules with numerous physiological functions in the field of biology, capable of performing functions such as energy storage and signal transmission. The MAMs is vital for lipid metabolic processes and the communication at the ER-mitochondria, as illustrated in Fig. 2(B). MAMs creates a hydrophilic region that facilitates the bidirectional noncystic transport process of lipids between the ER and mitochondria [63]. Despite many lipids metabolism-related enzymes are located in mitochondrial membranes, mitochondria are incapable of producing lipids from scratch. They initially depend on the ER, under the synergistic effect of MAMs, and synthesize the necessary lipids [64]. The increasing amount of research demonstrates that one of the MAMs’ primary roles is to regulate biosynthesis and intermembrane trafficking of phospholipid [65]. In the ER, phosphatidylserine synthases 1 (PSS1) and PSS2 catalyze the conversion of phosphatidic acid (PA) to phosphatidylserine (PS), after which PS is carried by lipid transporters at the contact sites to the cell membrane and mitochondria [66, 67]. Subsequently, phosphatidylethanolamine (PE) within mitochondria can be biosynthesized at the IMM by means of the PS decarboxylation pathway mediated by phosphatidylserine decarboxylase 1/2 (PSD1/2) [68]. The PS transport process from the ER to mitochondria is the key limiting factor determining the synthesis rate of PE. A portion of the PE produced by the mitochondria is immediately transported back to the ER, where it is modified to phosphatidylcholine (PC) by phosphatidylethanolamine N-methyltransferase (PEMT) [69]. Oxysterol-binding protein-related protein 5 (ORP5) and ORP8, as key proteins for lipid transport at the ER-mitochondrial contact site, interact with PTPIP51 or with the mitochondrial intermembrane space bridging (MIB)/mitochondrial contact sites and cristae junction organizing system (MICOS) complexes, mediating the non-cystic transport of PS from the ER to mitochondria [39, 70]. Guyard et al. observed that ORP5/8, in collaboration with the ER protein “seipin”, contributes to biogenesis of lipid droplet [71]. When PSS1 and PSS2 are knockdown, the level of PS declined, leading to a change in the homeostasis of PS in the ER and a shift in lipid synthesis towards diacylglycerol and triacylglycerol synthesis, in turn aggravating accumulation of lipid [67]. In the study of María Isabel et al, it was verified that MFN2 could bind to PS specifically and transport it to the mitochondria [72]. MFN2 knockdown lowered PSS1 expression and PS transfer from the ER to the mitochondria, degrading phospholipid synthesis and triggering ER stress [72]. Furthermore, it was shown that suppressing PSD activity significantly accelerated accumulation of PS in MAMs [73]. Additionally, the removal of PEMT declines the PC/PE ratio in the ER and stimulates ER stress [74]. Cardiolipin (CL), a characteristic lipid also present in the mitochondria, is necessary for both the triggering of apoptosis and mitochondrial bioenergetics [75]. The precursor PA for mitochondrial CL synthesis is located in the ER and transported to the mitochondria through the VAPB-PTPIP51 complex of the MAMs platform [76, 77].

Moreover, the MAMs are membrane domains containing cholesterol and related enzymes, often known as intracellular lipid rafts [78]. MAMs-associated caveolin-1 (CAV1) plays a crucial role in regulating the ER-mitochondrial cholesterol transport, and CAV1 manages cholesterol efflux by interacting with VDAC2 [29]. Silencing CAV1 is linked to aberrant intracellular deposits of free cholesterol as well as decreased physical integrity and extension of the MAMs [79]. The MAMs enzyme acetyl-coA cholesterol acyltransferase (ACAT1) catalyzes free cholesterol to form cholesteryl esters, thereby mediating cholesterol homeostasis [80]. The ER chaperone GRP78 regulates the folding state of the MAMs-related steroidogenic acute regulatory protein (StAR), ensuring the high activity of StAR in MAMs [81]. GRP78 interacts with StAR to form the cholesterol complex of MAMs, which then binds to the Ca^2+^-related protein VDAC2 located in MAMs to transport the substrate cholesterol to mitochondria for initiating steroidogenesis via OMM translocase [82, 83]. When VDAC2 is absent, StAR fails to transport cholesterol to the mitochondria [84]. Thus, the ER-mitochondrial region acts as a crucial center for steroidogenesis and metabolic initiation. MAMs also contributes to the production and transformation of ceramides, which is an important bioactive sphingolipid [85, 86]. MAMs contain various enzymes required for ceramides biosynthesis, such as ceramide synthase (CerS), ceramide glucosyltransferase (Cer-Glc-T), and sphingomyelin phosphodiesterase (SMase) [85, 87, 88]. The ceramide transfer (CERT) proteins in MAMs are responsible for transporting the ER ceramides to mitochondria [87]. Except for maintaining the lipid homeostasis of organelles, the MAMs is also vital in managing systemic lipid flux [89, 90].

### Mitochondrial dynamics

Mitochondria are highly dynamic structures composed of OMM, IMM, mitochondrial matrix and the intermembrane space, which can regulate cellular metabolism and energy synthesis [91, 92]. Accumulated evidence indicates that various membrane proteins residing on the inner and outer membranes play critical roles in modulating mitochondrial quality control (MQC) and MAMs activity, as shown in Fig. 2(C) [93, 94]. Mitochondrial dynamics, which primarily includes mitochondrial fusion, fission and motility, is an indispensable part of the MQC machinery [95, 96]. Mitochondrial dynamic balance maintains mitochondrial quality by regulating the ratio of fission and fusion. Recent studies have indicated that the biological process of mitochondrial dynamics may be impacted by the changes in MAMs.

Mitochondrial fusion can fix dysfunctional mitochondria and facilitate mitochondrial DNA (mtDNA) exchange, merge the contents of different mitochondria, and rescue defective mitochondria to retrieve vital components [97]. The mitochondrial fusion procedure mainly involves OMM fusion and IMM fusion [98]. Firstly, MFN2 on the surface of the ER can form dimers with MFN1 and MFN2 proteins on mitochondria, driving OMM fusion [99]. Then, IMM fusion depends on optic atrophy protein 1 (OPA1) [100]. Although MFN1 is exclusively located in mitochondria, MFN2 is enriched in MAMs, indicating that the connection between the ER and mitochondria contributes to mitochondrial fusion [35].

Mitochondrial fission is an essential component of mitochondrial dynamics and is crucial in modulating mitochondria quality and energy metabolism [101]. The dynamin-related protein 1 (DRP1), a type of GTPase in the dynamin protein superfamily, is the executor that propels mitochondrial fission [102]. Once dynamically recruited onto mitochondrial and peroxisome membranes, DRP1 polymerizes into helical oligomers and initiates membrane constriction in a GPT-dependent mode, eventually splitting the mitochondrial membrane [103, 104]. Remarkably, several specific adapter proteins enriched in MAMs, like FIS1, mitochondrial fission factor (MFF), and mitochondrial dynamic proteins 49 and 51 (MiD49/MiD51), must be present for the interaction between DRP1 and OMM [26, 105]. Since DRP1 is mostly distributed in the cytoplasm and fails to contain a domain that directly binds to membrane phospholipids, a group of MFF, MiD49 and MiD51 can be targeted to OMM by means of the receptor protein Fis1 [106]. Furthermore, the ER-localized inverted formin 2 (INF2) mediates actin polymerization at MAMs, stimulating an increase in mitochondrial Ca^2+^ uptake, which is a key step in mitochondrial division [107, 108]. Several other MAM-related proteins were also found to be involved in the mitochondrial fission process, including FUNDC1 and phosphofurin acidic cluster sorting protein 2 (PACS2) [109, 110]. FUNDC1 interacts with ER-resident calnexin (CANX) in the hypoxic environment and accumulates in MAMs. Once mitochondria undergo autophagy, FUNDC1-CANX will dissociate and recruit DRP1 to facilitate the occurrence of mitochondrial fission [111]. Li et al. found that the overexpression of PACS-2 inhibits mitochondrial fission by blocking DRP1 [110].

Mitochondrial motility is carried out along microtubules through the action of opposing kinesin and dynein motors, thereby regulating the distribution of mitochondria in the cytoplasm and meeting the normal physiological demand of the cell [112]. Dynein and kinesin are not directly linked to OMM, but indirectly associate with OMM proteins MIRO1 and MIRO2 through mitochondrial adapter proteins (TRAK1 and TRAK2) [113]. It has been reported that MIRO1 and MIRO2 are normal mitochondrial crista architecture and their deletion alters the ER-mitochondrial contact sites [114]. The roles of MIRO1 and MIRO2 in mitochondrial motility is intimately connected with MAMs. Hence, MAMs may be involved in mitochondrial motility.

### Endoplasmic reticulum stress

The ER is responsible for protein synthesis, folding, post-translational modification and secretion, serving as a quality organelle for controlling protein homeostasis [115]. ER stress occurs in response to hypoxia, metabolic disorders, and oxidative stress, giving rise to the accumulation of misfolded and unfolded proteins within the ER lumen and then disrupting proteostasis [116]. Initiating the adaptive unfolded protein response (UPR) contributes to rebuild the ability of folding proteins to restore ER homeostasis [117]. Inositol-requiring enzyme 1α (IRE1α), activating transcription factor 6 (ATF6), and protein kinase RNA-like endoplasmic reticulum kinase (PERK) are three key transmembrane sensor proteins, which can detect the accumulation of unfolded or misfolded proteins and trigger UPR [118]. It has been shown that UPR sensors have been identified in MAMs, and the relationship between MAMs and ER stress becomes unambiguous [Fig. 2(D)]. PERK, as the most conserved ER stress sensor, is biologically linked to the key protein MFN2 of MAMs [119]. MFN2 is an upstream inhibitor of PERK, which can prevent the transfer of reactive oxygen species ‌(ROS) to mitochondria caused by excessive activation time of PERK, thereby avoiding the resulting changes in mitochondrial morphology and function, and ultimately playing a role in maintaining the homeostasis of the ER [120]. Additionally, IRE1 is predominantly present at the MAMs, where IRE1 promotes dimerization by physically interacting with the ER chaperone Sig-1R during cellular ER stress [121]. Excessive activation of IRE1α can inhibit apoptosis caused by endoplasmic reticulum stress [122]. When ER stress is initiated, ATF6 undergoes a series of sophisticated biological processes and is transferred to the nucleus, activating the UPR [123]. It was also discovered that VAPB enriched in the ER not only binds to PTPIP5 but also directly interacts with ATF6, thereby reducing its activity [124].

### Glucose homeostasis

**Fig. 3:**
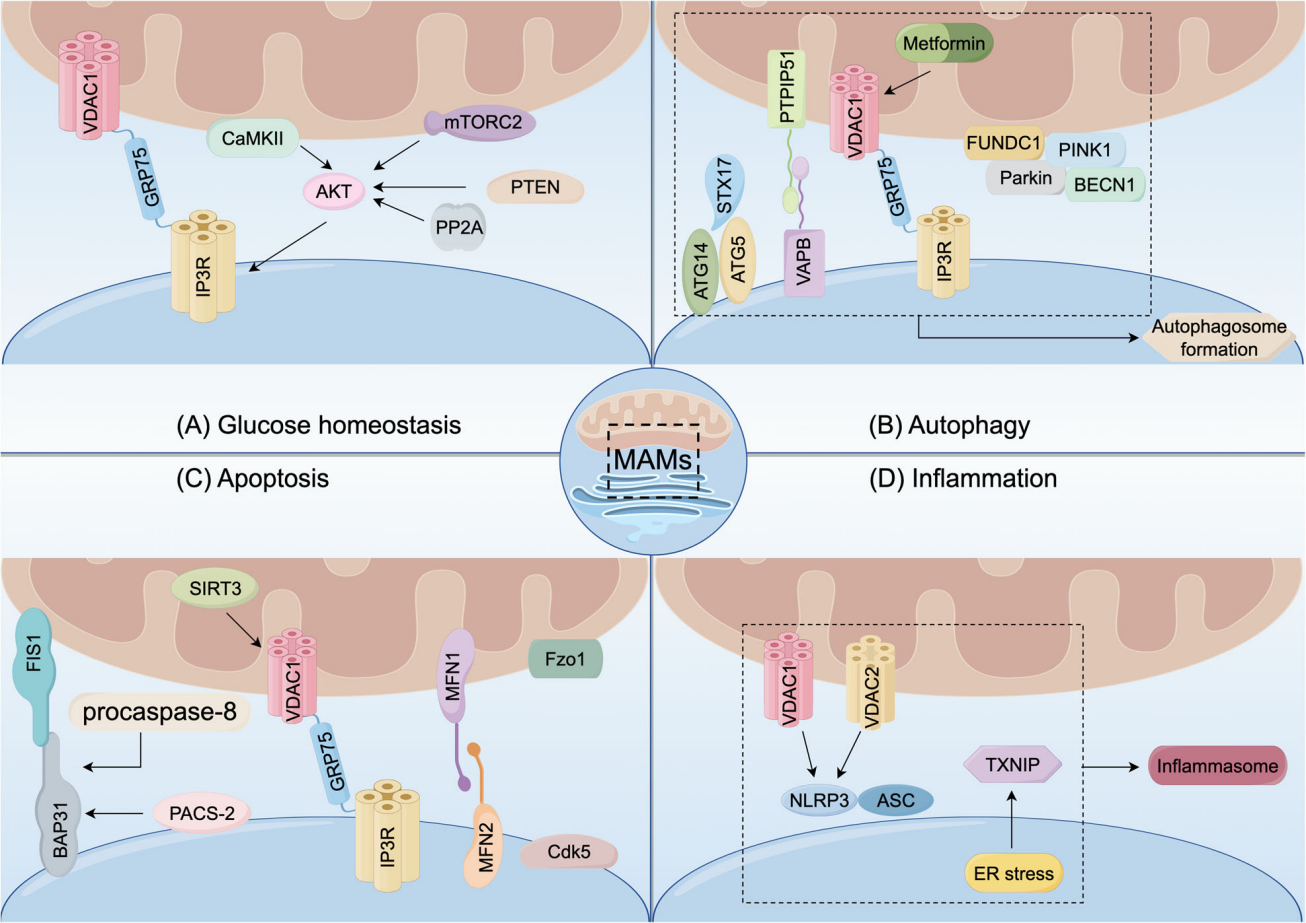
The biological functions of MAMs. This figure illustrates the biological functions of MAMs, including (**A**) glucose homeostasis; **B** autophagy; **C** apoptosis; **D** inflammation.

Glucose metabolism, as the core hub for energy supply and material synthesis in organisms, achieves a dynamic balance of glucose within the body through a series of biochemical processes. The insulin pathway is one of the key mechanisms to regulate glucose homeostasis, which maintains glucose homeostasis by coordinating biological processes such as peripheral uptake, hepatic glucose control, and energy storage [125]. The MAMs recruit proteins implicated in the insulin signaling pathway, suggesting a potential relationship between MAMs and insulin signaling [Fig. 3A] [126]. After insulin binds to the membrane receptor, it activates the downstream signal Akt through a series of reactions [10]. Akt, located on MAMs, effectively inhibits the release of Ca^2+^ and the initiation of apoptosis by driving the phosphorylation of IP3R [127]. It has been demonstrated that elevated cytoplasmic Ca²⁺ levels can lead to hyperphosphorylation of calmodulin-dependent protein kinase II (CaMKII) and is involved in the progression of insulin resistance [128]. Ozcan et al. reported that CaMKII affects insulin signaling by enhancing Akt phosphorylation [129]. It was observed in pancreatic β-cell that knockout of CaMKII resulted in glucose-stimulated insulin secretion dysfunction [130]. mTORC2 is also localized to MAMs, where it controls the integrity of MAMs by phosphorylating and activating the kinase Akt [131]. In addition, it was found that the phosphatase and tensin homolog (PTEN) and the protein phosphatase 2 A (PP2A) were also enriched in MAMs and involved in Ca^2+^ transfer by regulating Akt phosphorylation and IP3R [127, 132]. Thus, the mitochondrial-ER contact site plays an indispensable role in modulating the insulin pathway, thereby supporting the influence of MAMs on glucose metabolism. Although current research has confirmed that MAMs are involved in insulin signaling, it is still at the initial stage of mechanism exploration and further studies are needed to deeply analyze the molecular mechanism by which MAMs regulate glucose metabolism through the insulin pathway.

### Autophagy

In eukaryotes, autophagy, as a highly conserved decomposition mechanism, can not only specifically eliminate misfolded proteins or damaged components, but also initiate a non-selective degradation mode in response to nutrient deficiency [133]. In the process of autophagy, the autophagosome forming a bilayer membrane structure fuses with the lysosome and precisely acts on the content to degrade it. The dynamic interaction between autophagy and MAMs is intimate and complicated, as shown in Fig. 3(B). Under starvation conditions, it was found that autophagosome markers ATG14 and ATG5 were located at MAMs, and ATG14 was effectively recruited to the MAMs site by the SNARE protein syntaxin 17 (STX17). Moreover, the disruption of the ER-mitochondria site can inhibit the recruitment of ATG14 [134]. The binding chain formed by the mitochondrial protein PTPIP51 and the endoplasmic reticulum protein VAPB is essential for the formation of autophagosomes. Gomex-suaga et al. reported that the absence of VAPB-PTPIP51 relaxes the contact of MAMs to drive autophagosome formation, while the overexpression of VAPB-PTPIP51 tightened this site and prevented autophagosome formation [38]. Minjeong et al. discovered that metformin facilitates autophagy by binding to the MAMs anchor protein VDAC1 and disrupting the integrity of the IP3R-GRP75-VDAC1 complex [135]. As a selective autophagy process, mitophagy can maintain the quality stability of the mitochondria and cellular homeostasis [136]. Key factors of mitophagy, such as PTEN induced kinase 1 (PINK1), Beclin 1 (BECN1), Parkin, and FUNDC1, are enriched at the MAMs site, promoting the formation of autophagosomes to degrade damaged mitochondria [111, 137, 138].

### Apoptosis

When cells are unable to effectively respond to various stresses, the apoptotic pathway is initiated, and this programmed death can orderly remove damaged cells [139]. Apoptosis is intimately associated with the structural and functional alterations of the ER-mitochondrial site, as can be seen in Fig. 3C. The mitochondrial outer membrane receptor Fis1 regulates mitochondrial fission events, while the cleavage of the ER protein BAP31 affects apoptosis related to membrane rupture [140, 141]. The Fis1-Bap31 complex, which connects the mitochondrial-ER contact site, induces the activation of apoptotic signaling. The recruitment of procaspase-8 by the Fis1-Bap31 complex is an early apoptotic event [34]. The variation in the death effector domain of Bap31 is related to procaspase-8 and is also the core of activating procaspase-8, thereby stimulating the release of Ca^2+^ from the ER and facilitating mitochondrial apoptosis. It was also found that PACS-2 controls the stable communication between mitochondria and ER, and its deletion triggers BAP31-dependent mitochondrial fragmentation and activation of the apoptotic signal caspase-3 [142]. In addition, the MAMs protein complex IP3R-GRP75-VDAC1 is involved in apoptosis signaling by regulating calcium levels [143]. SIRT3 has been demonstrated to attenuate Ca^2+^ overloading-mediated apoptosis by reducing the VDAC1-GRP75-IP3R complex and interfering with the formation of MAMs [144]. Saranya et al. found that the loss of Cdk5 located in MAMs leads to an imbalance in mitochondrial Ca^2+^ homeostasis, induces the opening of mitochondrial permeability transition pores, and further promotes apoptosis [145]. Besides, some proteins involved in mitochondrial fusion/fission, such as MFN1/2 and Fzo1, are associated with apoptosis [146].

### Inflammation

A dynamic link between inflammation and MAMs has been demonstrated, and the related molecular mechanisms are shown in Fig. 3D [147]. Currently, the NLRP3 complex is the most thoroughly studied MAMs-related inflammasome. Usually, NLRP3 is located on the ER membrane, but once activated, both NLRP3 and its important component ASC will be translocated to the MAMs contact site [147]. This process found an increase in ROS produced by damaged mitochondria. Under NLRP3 activation or oxidative stress conditions, the thioredoxin interacting protein (TXNIP) of NLRP3 will also be located in the MAMs [148]. In addition, TXNIP can be activated under the ER stress through PERK and IRE1 [149]. It has been revealed that the NLRP3-mediated inflammatory response is also strongly associated with the VDAC protein located in MAMs. Silencing VDAC1 and VDAC2 significantly downregulated the levels of NLRP3 inflammasomes compared with VDAC3 [147].

## Mams and diabetic microvascular complications

### MAMs and diabetic kidney disease

**Fig. 4:**
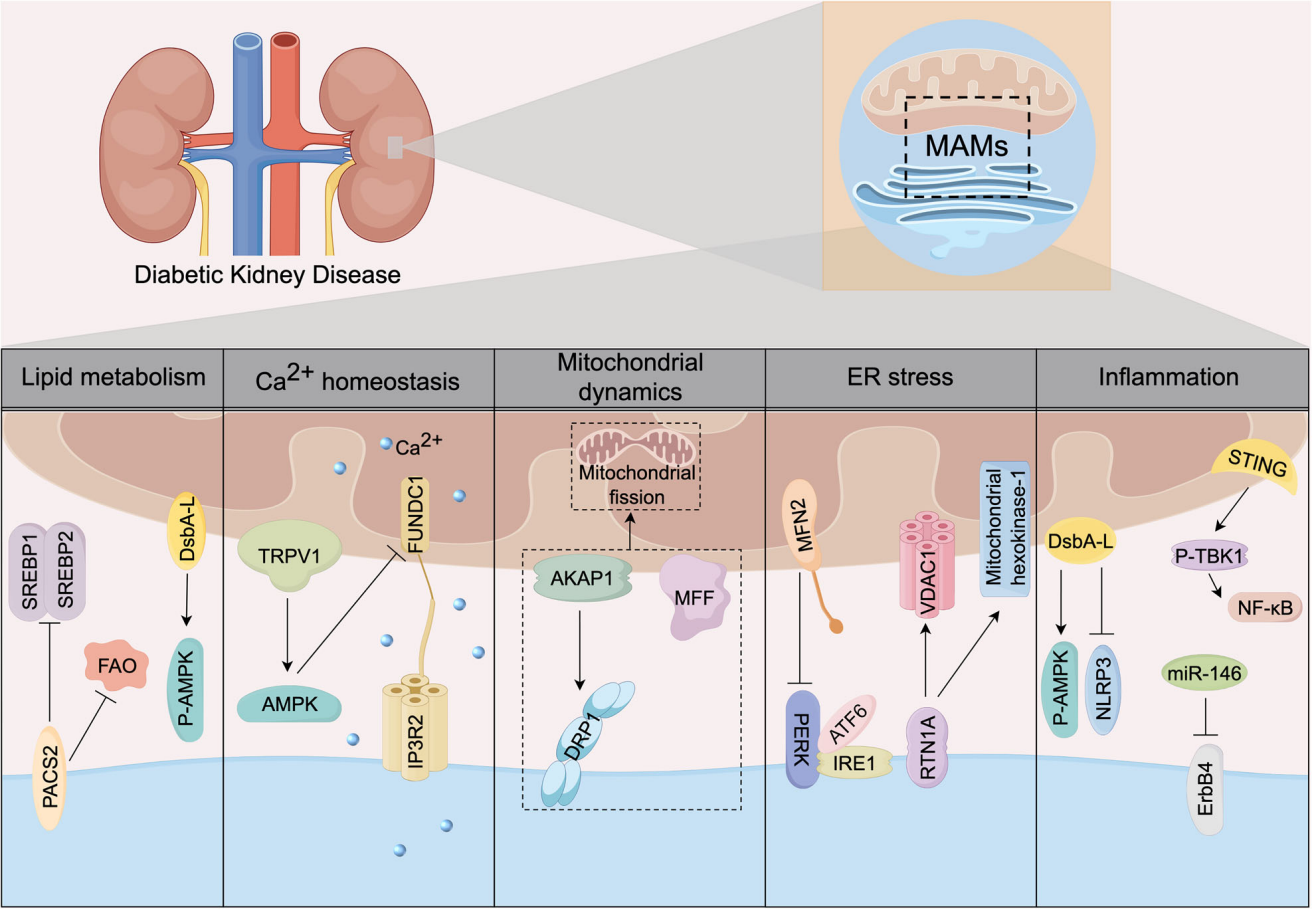
The multiple roles of MAMs in DKD. MAMs are special contact sites between the ER and mitochondria. MAMs resident proteins play a key role in regulating the development of DKD, including lipid metabolism, Ca^2+^ homeostasis, mitochondrial dynamics, ER stress, and inflammation.

DKD is the renal microvascular complication caused by diabetes, characterized by progressive decline in renal function and persistent proteinuria [150, 151]. DKD has emerged as the most common and serious diabetes complication, and it is the primary cause of end stage renal disease (ESRD) [152]. By 2045, the number of diabetes patients is expected to rise to 700 million, among which approximately 36% of DM patients will be combined with DKD, and 50% of DKD patients will deteriorate to ESRD [153, 154]. The main pathological changes of DKD include thickening of the glomerular basement membrane (GBM), glomerulosclerosis and interstitial fibrosis. In recent years, the crucial role of organelles in human diseases has received increasing attention. With the in-depth exploration of organelles, it has been found that MAMs, as a dynamic contact area between mitochondria and the ER, are closely related to the progression of DKD [155]. MAMs-related proteins regulate numerous cellular processes, including lipid metabolism, calcium homeostasis, mitochondrial fusion and fission, ER stress, inflammation and autophagy, as described in Fig. 4.

Lipid metabolism disorder is the signature pathological feature of DKD. It has been reported that the expression of MAMs related proteins such as PACS2, DsbA-L, and MFN2 is decreased and negatively correlated with the degree of renal lipid deposition [156]. In diabetic mice, renal tubular-specific knocking down of PACS2 elevated the levels of sterol regulatory element binding protein (SREBP) 1/2 and aggravated renal lipid deposition, indicating that PACS2 deficiency in DKD inhibits lipid metabolism in the kidneys [157]. Furthermore, PACS2 is involved in the pathological damage of renal vascular hyperpermeability and glomerular sclerosis in DKD mice via the regulation of fatty acid β-oxidation metabolism [158]. Chen et al. found in the study that disulfide-bond A oxidoreductase-like protein (DsbA-L) might have a significant protective effect on lipid-related renal damage in DKD by activating the AMPK pathway [159].

The imbalance of calcium homeostasis is involved in the pathogenesis of intrinsic cell damage in DKD [160]. Excessive mitochondrial calcium intake leads to impaired protein filtration function of podocytes, which is an important pathological feature of DKD. Wei et al. found in their study that dietary capsaicin reverses the damaged renal structure and function in diabetic mice in a way that depends on the transient receptor potential cation channel subfamily V member 1 (TRPV1) of Ca^2+^ channels [161]. Mechanistically, TRPV1 decreases the transport of Ca^2+^ from the ER to mitochondria by activating AMPK and suppressing the transcription of FUNDC1 [161].

Under physiological conditions, the equilibrium of mitochondrial dynamics is crucial for cell survival and bioenergy metabolism, and is closely related to kidney function. Before the occurrence of proteinuria and renal pathological changes in DM, mitochondrial dynamics and ultrastructure have already changed, and these changes in the mitochondria also occur with the progression of DKD [162]. The regulation of mitochondrial division and fusion is mediated by mam-related proteins, including DRP1, MFF, MFN1, MFN2 and OPA1. By modulating DRP1 phosphorylation and its subsequent mitochondrial translocation, the increased transport of A-kinase-anchored protein 1 (AKAP1) to MAMs facilitated podocyte mitochondrial fission [163]. Xiao et al. reported that MFF expression was positively correlated with podocyte mitochondrial division under high glucose conditions [164].

In DKD, the phenomenon of ER stress and protein misfolding are significant [165]. Three key transmembrane proteins, IRE1α, ATF6 and PERK, regulate unfolded or misfolded proteins and activate the UPR. The MAMs also contains numerous ER partners that are involved in protein folding, such as Sig1R, Bid, calnexin, calreticulin [166]. Cao et al. observed that IRE1, ATF6 and PERK were activated in DKD rats, while the expression of Mfn2 and podocyte MAMs decreased [167]. Xie et al. found that the specific overexpression of ER protein RTN1A aggravated ER stress and mitochondrial dysfunction in renal tubular epithelial cells under diabetic conditions by regulating ER-mitochondrial contact [155]. And this phenomenon is caused by the physical interaction of RTN1A with VDAC1 and mitochondrial hexokinase-1 [155].

The activation of inflammatory pathways is inseparable from the pathogenesis of DKD [168]. The interaction between NLRP3 inflammasome and MAMs protein in DKD may aggravate renal injury. A recent study pointed out that DsbA-L interferes with the activation of NLRP3 inflammasome by stimulating the phosphorylation of AMPK in HK-2 cells induced by high glucose, thereby alleviating renal injury [169]. Furthermore, interferon gene-stimulating factor (STING), as a mediator of innate immunity, has been proven to be located in MAMs [170]. STING was found to accelerate podocyte injury in db/db mice [171]. Additionally, it was shown that MAMs are subcellular locations of specific miRNAs, such as miR-146. Importantly, miR-146 are involved in regulating the inflammatory response of DKD [172]. Thus, there is a possibility that MAMs inhibit renal inflammation in DKD via miRNAs.

### MAMs and diabetic cardiomyopathy

DCM is a chronic cardiovascular complication of DM, directly associated with the progression of heart failure, and is also a key factor for the death of diabetic patients [173]. Its typical pathological feature is the abnormality of ventricular structure and function, mainly manifested as left ventricular hypertrophy and early-onset diastolic dysfunction, with or without systolic dysfunction [174]. DCM is an irreversible process and is independent of hypertensive heart disease and coronary artery disease [175]. The pathogenesis of DCM is multifactorial and unclear, and currently there is a lack of effective treatment strategies. Recent studies have shown that the destruction of MAMs sites is the key contributing factor in the progression of cardiovascular diseases [176].

**Fig. 5:**
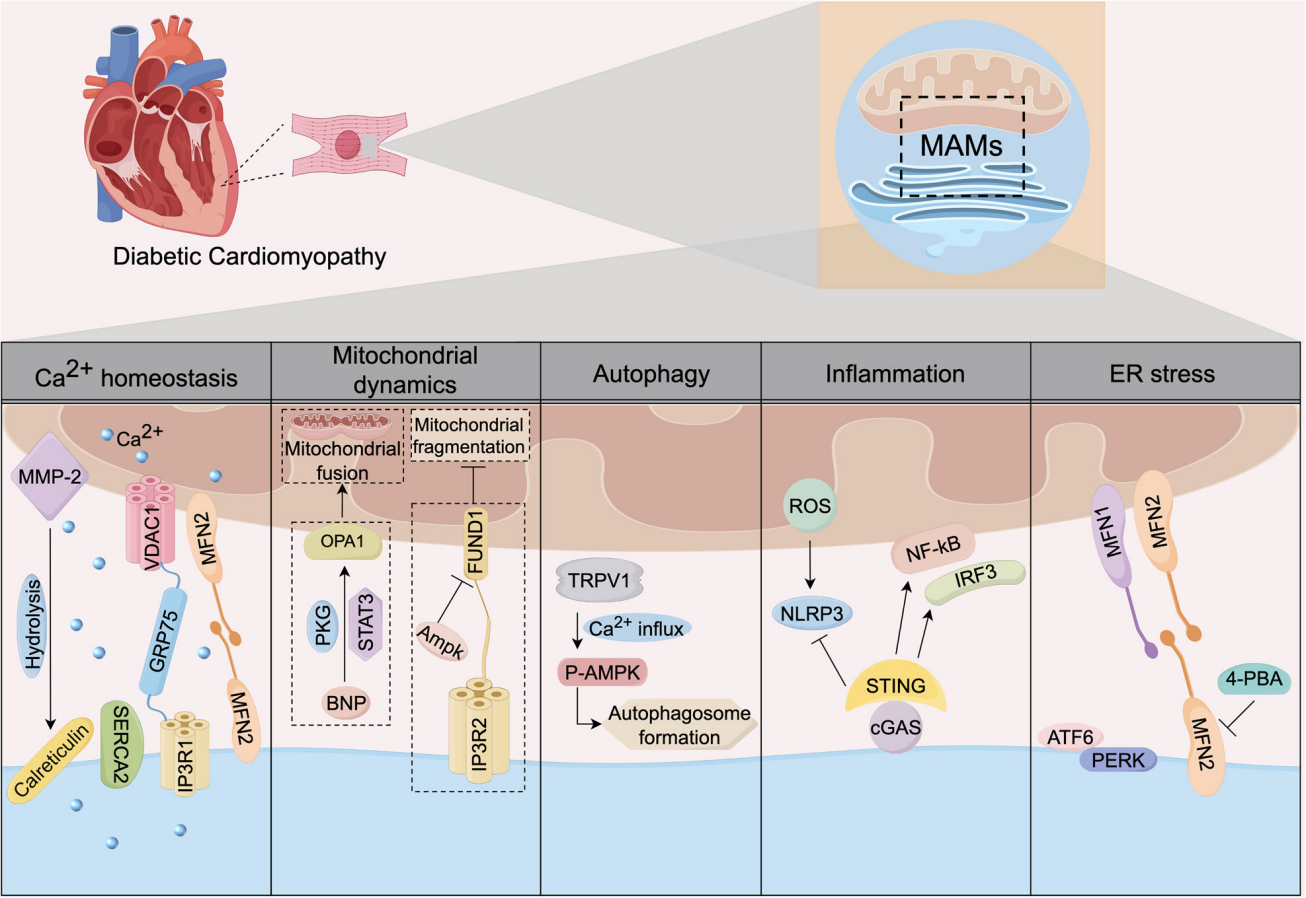
MAMs mediate multiple cellular processes in DCM. The resident proteins of MAMs regulate various cellular processes related to the progression of DCM, including Ca^2+^ homeostasis, mitochondrial dynamics, autophagy, inflammation, and ER stress.

MAMs have also been confirmed to be directly or indirectly involved in the progression of DCM [177]. Under high glucose conditions, the destruction of MAMs integrity in cardiomyocytes in DM environments leads to imbalances in various biological processes, which could account for the metabolic abnormalities in DCM (Fig. 5).

The interference of Ca^2+^ signals in MAMs is closely related to cardiac dysfunction in DCM. Matrix metalloproteinase-2 (MMP-2), mainly distributed in MAMs of cardiomyocytes, can regulate Ca^2+^ homeostasis by modulating the expression of calreticulin [178]. In DCM, it was observed that the down-regulation of SERCA2 protein expression caused changes in Ca^2+^ and cardiac contractility, affecting the progression of DCM [179]. Silencing GRP75 in cardiomyocytes (HL-1 cells) resulted in impaired transport of calcium from ER to mitochondria, thereby alleviating calcium overload and mitochondrial oxidative stress [180]. Yuan et al. discovered that the silencing of Mfn2 in HL-1 cells alleviated Ca^2+^ overload and oxidative stress in mitochondria, thereby exerting a protective effect on atrial cardiomyocytes [181].

The abnormality of mitochondrial function is the key to the pathogenesis of DCM. Morphological measurement analysis indicated that diabetic cardiomyocytes had a smaller mitochondrial size and greater spatial density compared to control cardiomyocytes, thereby enhancing the availability of ATP in the cytoplasm and generating a moderate compensatory effect [182]. A recent study has found that elevated BNP levels facilitate OPA1-mediated mitochondrial fusion, maintain mitochondrial respiration, and delay the progression of DCM [183]. Mechanistically, BNP prevents hyperglycemia-induced mitochondrial oxidative damage and DCM by activating the NPRA-PKG-STAT3-OPA1 pathway [183]. Additionally, it was found that STAT3 is not located in the mitochondria but in the MAMs part [183]. Specific knockout of FUNDC1 by cardiomyocytes can eliminate the formation of MAMs induced by diabetes, prevent mitochondrial fragmentation and mitochondrial calcium overload, suggesting that inhibition of FUNDC1 is a potential target of DCM [184].

Autophagy is a double-edged sword, and maintaining an appropriate level of autophagy is crucial for cardiomyocyte homeostasis. The cardiac function of DCM can be impacted by either excessive or insufficient autophagy [185]. The study has shown that maintaining myocardial autophagy homeostasis depends on the level of Ca^2+^. Wei et al. found that TRPV1 could trigger the influx of Ca²⁺, phosphorylate AMPK, and promote the autophagy process of cardiomyocytes [186]. In addition to Ca^2+^, MAMs also mediate autophagy through mitochondrial homeostasis, lipid metabolism and other pathways.

The main characteristics of DCM include persistent myocardial inflammation. Blocking the activation of NLRP3 inflammasome is a potential target for delaying the progression of DCM. Once NLRP3 is activated and transported to MAMs, it subsequently interacts with the adapter apoptosis-associated speck-like protein containing a caspase recruitment domain (ASC) [187]. It was also found that STING located in MAMs can regulate the activity of NLRP3 [188]. Ma et al. found that the cyclic GMP-AMP synthase (cGAS)/STING signaling pathway and its downstream targets IRF3, NF-κB, IL-18 and IL-1β were activated in the DCM mouse model [189]. In addition, inhibiting STING significantly alleviates the inflammatory response and apoptosis of cardiomyocytes and delays DCM [189].

ER stress is an early event related to DCM and may be triggered by several factors such as hyperglycemia, free fatty acids, insulin resistance and inflammation [190]. These pathological conditions will also further aggravate ER stress [191]. In DCM rats and the H9C2 cell model stimulated by high glucose, inhibiting the expression of ATF6 and PERK in the ER stress pathway can attenuate the apoptosis of cardiomyocytes [192]. In addition, silencing Mfn2 was found to prevent mitochondrial dysfunction and ER stress in high glucose-induced atrial cardiomyocytes, thereby improving cardiomyocyte death [181].

### MAMs and diabetic retinopathy

**Fig. 6:**
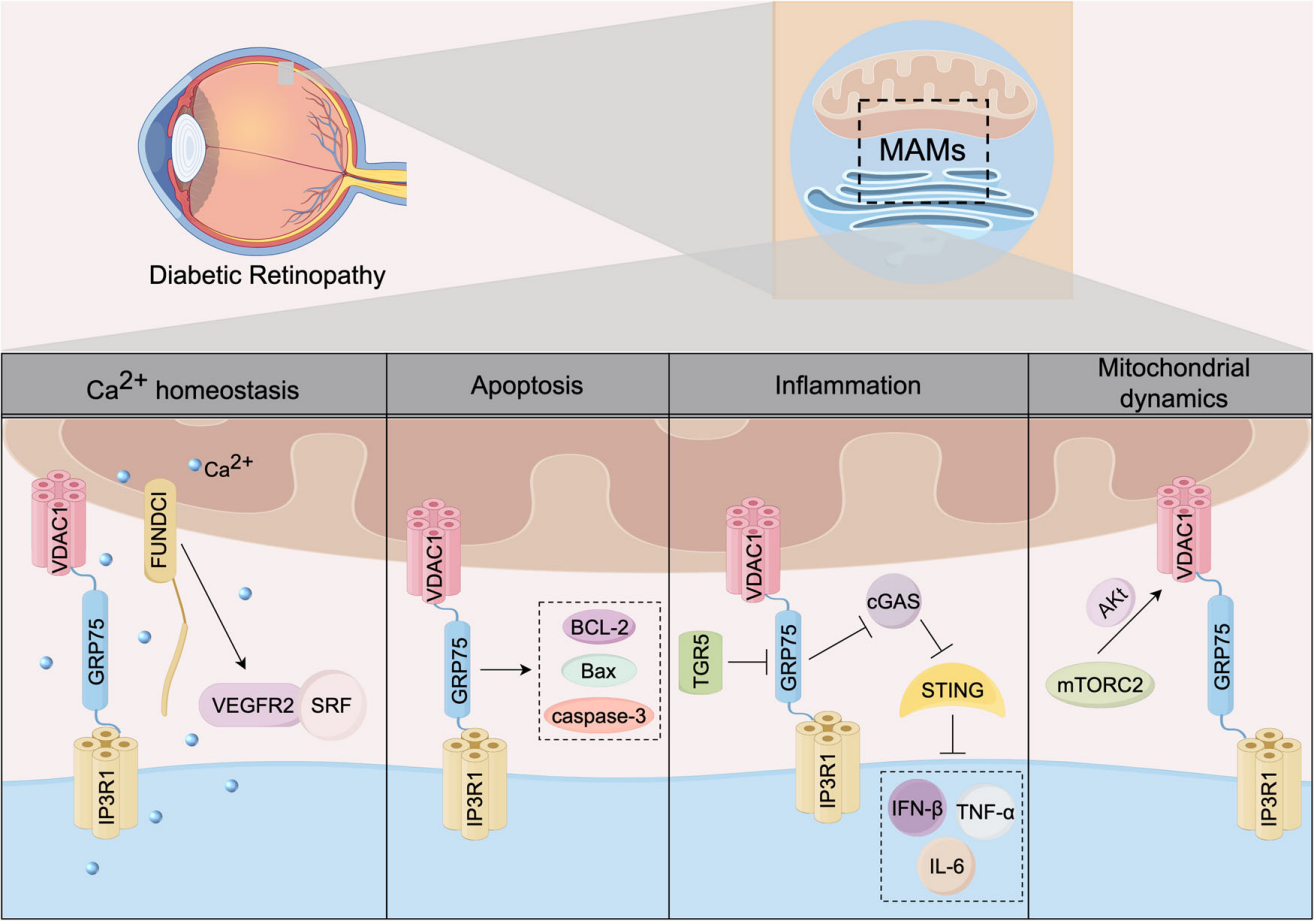
The functional description of MAMs in DR. The proteins on MAMs have a regulatory effect on the progression of DR, and the involved cellular processes include Ca^2+^ homeostasis, apoptosis, inflammation, and mitochondrial dynamics.

DR is one of the common and specific microvascular complications of diabetes and is a significant global health problem [193]. Globally, the prevalence of DR among DM patients was 22.27%, and it is expected to reach 55.6% by 2045. In the early stage of DR, patients have no obvious symptoms. As the disease progresses, they may experience blurred vision, visual field defects, and in severe cases, retinal detachment, leading to varying degrees of vision loss or even blindness [194]. The fundus lesions of DR include microhemangioma, petechiae, hard exudate and neovascularisation. Without timely intervention, irreversible visual impairment might appear in the later stage, which will have a serious impact on the quality of life of DM patients. Recent studies have emphasized the significance of targeting MAMs in the pathogenesis and treatment of eye diseases. The role of MAMs in DR is depicted in Fig. 6.

Retinal microvascular endothelial cells (RMECs) are regarded as classic pathological targets in DR. According to research findings, mitochondrial dysfunction mediated by Ca^2+^ overload could result in the accumulation of ROS, which in turn triggers RMEC apoptosis. Li et al. found that the activation of the IP3R1-GRP75-VDAC1 axis promotes the formation of MAMs sites and the transport of Ca^2+^ from the ER to mitochondria, accelerating the elevation of mitochondrial ROS levels and the occurrence of apoptosis both in vivo and in vitro model [195].

In proliferative diabetic retinopathy (PDR), the formation of neovascularization is regulated by vascular endothelial growth factor (VEGF) and its receptor VEGFR [196]. One study found that the disruption of MAMs formation led to a decrease in Ca^2+^ in the cytoplasm, inhibited the phosphorylation of serum response factor (SRF), reduced the interaction between SRF and the VEGFR2 promoter, and weakened the expression of VEGFR2, thereby blocking angiogenesis [197]. However, the occurrence of these biological phenomena is attributed to the specific loss of FUNDC1 in endothelial cells [197].

Chronic inflammation is also one of the early manifestations of DR lesions. Muller cells, as the primary target cells of neuroinflammation, run through the entire retina to preserve the homeostasis of the retinal neurovascular unit [198]. Li et al. found that down-regulating the expression of the IP3R1-GRP75-VDAC1 axis could alleviate mitochondrial Ca^2+^ overload in Murell cells, inhibit the activation of mtDNA-mediated cGAS-STING, and thereby ameliorate the retinal neuroinflammatory response [199]. Furthermore, the knockdown of TGR5 can reverse these changes and aggravate retinal damage in DR [199].

The target of rapamycin (TOR), as a highly conserved protein kinase, is involved in cell growth and proliferation. Mammalian TOR complex 2 (mTORC2) is located in MAMs under the stimulation of growth factors and maintains the stability of MAMs and mitochondrial function by AKT-mediated MAM-related proteins [131]. Studies have found that retinal pigment epithelial cells (ARPE-19) treated with high glucose will undergo a series of physiological and pathological changes, including mitochondrial dysfunction, inflammatory response and oxidative stress [200–203]. However, in the context of diabetic retinopathy, the mTOR signaling pathway in MAMs is still an area of ongoing exploration.

## Conclusion

MAMs serve as the key platform for substance transfer and signal transduction in the physiology and pathology of DMC. Any stimulation that damages mitochondria and ER will affect the integrity of MAMs, and the alteration of MAMs in turn disrupts the structure and function of organelles, exacerbating the progression of diseases. Emerging evidence has shown that MAMs play a crucial role in regulating lipid metabolism, Ca^2+^ homeostasis, MQC, endoplasmic reticulum stress, autophagy and apoptosis, and inflammation. Based on the current research, several MAMs resident proteins have been thoroughly examined in DKD, DCM and DR, such as IP3R1, MFN2, FUNDC1 and PACS2.

Targeting MAMs, as a promising therapeutic measure, has attracted attention in recent years. Dietary capsaicin reduces the formation of MAMs and the transport of Ca^2+^ from ER to mitochondria in a TRPV1-dependent manner, alleviates mitochondrial dysfunction under hyperglycemic conditions, and reverses kidney injury in DM mice [161]. Mitochondria-targeted peptide SS31 improves mitochondrial structural damage and alleviates renal injury in DKD via increasing the expression of Mfn1 and inhibiting the expression of Drp1 [204]. Besides, metformin, Wogonin and curcumin have also been confirmed to target MAMs for the treatment of DKD [205–207]. Exendin-4, an agonist of the glucagon-like peptide-1 receptor (GLP-1R), has been shown to lessen mitochondrial oxidative stress in diabetic hearts. Yunce et al. found that Exendin-4 alleviated high glucose-induced cardiomyocyte apoptosis by enhancing SERCA2a activity and reducing ER stress [208]. Furthermore, MAMs-specific proteins MFN2 and FUNDC1 are highly expressed in cardiomyocytes and are involved in regulating mitochondrial fusion and Ca^2+^ homeostasis, which are expected to become potential targets for the treatment of DCM. However, given the high heterogeneity of MAMs, effective strategies for treating DR with MAMs face huge challenges.

So far, there are two questions regarding the mechanism of MAMs in DMC. Firstly, due to the dynamic nature of MAMs structure, it is necessary to apply emerging technologies, such as split GFP-based contact site sensor (SPLICS) constructs, which can monitor MAMs in real time and perform proteomic analysis to identify core pathways of organelles in disease. Secondly, the regulation of communication between mitochondria and the ER is twofold. The increase in the spatial distance between the ER and mitochondria promotes mitochondrial Ca^2+^ uptake and activates ATP synthesis, while mitochondrial Ca^2+^ overload leads to mitochondrial swelling and the release of pro-apoptotic factors. Hence, optimal regulation of MAMs structure and function can maintain the balance of microenvironment.
